# Viral Coinfection among COVID-19 Patient Groups: An Update Systematic Review and Meta-Analysis

**DOI:** 10.1155/2021/5313832

**Published:** 2021-09-03

**Authors:** Pooneh Malekifar, Reza Pakzad, Ramin Shahbahrami, Milad Zandi, Ali Jafarpour, Sara Akhavan Rezayat, Samaneh Akbarpour, Alireza Namazi Shabestari, Iraj Pakzad, Elahe Hesari, Abbas Farahani, Saber Soltani

**Affiliations:** ^1^Department of Epidemiology, School of Public Health, Tehran University Medical Sciences, Tehran, Iran; ^2^Department of Epidemiology, Faculty of Health, Ilam University Medical Sciences, Ilam, Iran; ^3^Student Research Committee, Ilam University Medical Sciences, Ilam, Iran; ^4^Research Center for Clinical Virology, Tehran University of Medical Sciences, Tehran, Iran; ^5^Department of Virology, School of Public Health, Tehran University of Medical Sciences, Tehran, Iran; ^6^Gerash Amir-al-Momenin Medical and Educational Center, Gerash University of Medical Sciences, Gerash, Iran; ^7^Department of Management & Health Economics, School of Public Health, Tehran University of Medical Sciences, Tehran, Iran; ^8^Occupational Sleep Research Center, Baharloo Hospital, Tehran University of Medical Sciences, Tehran, Iran; ^9^Department of Geriatric Medicine, School of Medicine, Tehran University of Medical Sciences, Tehran, Iran; ^10^Department of Microbiology, School of Medicine, Ilam University Medical Sciences, Ilam, Iran; ^11^Infectious and Tropical Diseases Research Center, Hormozgan Health Institute, Hormozgan University of Medical Sciences, Bandar Abbas, Iran

## Abstract

**Background:**

Coinfections have a potential role in increased morbidity and mortality rates during pandemics. Our investigation is aimed at evaluating the viral coinfection prevalence in COVID-19 patients.

**Methods:**

We systematically searched scientific databases, including Medline, Scopus, WOS, and Embase, from December 1, 2019, to December 30, 2020. Preprint servers such as medRxiv were also scanned to find other related preprint papers. All types of studies evaluating the viral coinfection prevalence in COVID-19 patients were considered. We applied the random effects model to pool all of the related studies.

**Results:**

Thirty-three studies including 10484 patients were identified. The viral coinfection estimated pooled prevalence was 12.58%; 95% CI: 7.31 to 18.96). Blood viruses (pooled prevalence: 12.48%; 95% CI: 8.57 to 16.93) had the most frequent viral coinfection, and respiratory viruses (pooled prevalence: 4.32%; 95% CI: 2.78 to 6.15) had less frequent viral coinfection. The herpesvirus pooled prevalence was 11.71% (95% CI: 3.02 to 24.80). Also, the maximum and minimum of viral coinfection pooled prevalence were in AMRO and EMRO with 15.63% (95% CI: 3.78 to 33.31) and 7.05% (95% CI: 3.84 to 11.07), respectively.

**Conclusion:**

The lowest rate of coinfection belonged to respiratory viruses. Blood-borne viruses had the highest coinfection rate. Our results provide important data about the prevalence of blood-borne viruses among COVID-19 patients which can be critical when it comes to their treatment procedure.

## 1. Introduction

The novel coronavirus outbreak started in Wuhan, China, and suddenly turned into one of the worst pandemics that have ever been experienced [1]. As of July 30, over 197 million infected persons and more than 4 million deaths were recorded [2]. Using different limitation methods to stop SARS-CoV-2 transmission, including national lockdown, was almost useless [3].

There are no practical clinical guidelines to manage the viral coinfection in COVID-19 patients, and their treatment takes place based on previous experience learned from other outbreaks including influenza and SARS [4].

SARS-CoV-2's extremely variable nature and insufficient information about host-pathogen interaction caused a great challenge to discover effective treatments against the virus [5]. Viral coinfection in COVID-19 patients may lead to more trouble in patient's recovery from the disease [6]. The interplay between different viruses and SARS-CoV-2 and their synergistic effect on disease clinical variation are under investigation [7].

The majority of respiratory viruses include respiratory syncytial virus human metapneumovirus and rhinovirus can cause coinfections; therefore, providing information about coinfection with these viruses in COVID-19 patients has a diagnostic value that can be helpful in the patients' outcome prediction [8]. It should be noted that providing data about coinfection with other respiratory viruses in COVID-19-confirmed cases could be crucial for patient antiviral therapy. Individuals with coinfections may show different reactions to treatment compared to those with only COVID-19 [9]. It is also considered that the synergy between COVID-19 and respiratory viruses could increase the disease progression. This matter is important especially among high-risk patients, including those with immunodeficiency and immunosuppression [10].

Several studies observed respiratory virus coinfection in COVID-19. About 5.8% of the confirmed COVID-19 cases in Wuhan were infected with other types of other respiratory viruses [11]. Another study in Northern California reported that the 20.7% positive COVID-19 specimens were infected with at least one more pathogens, and among them, respiratory viruses were the most coinfected agents [8]. Although based on different reports, the respiratory virus coinfection prevalence among COVID-19-infected people is surprisingly low. It is quite clear that enough information about the viral coinfection prevalence and types of viruses will help the clinician to run a better diagnosis and treat patients more precisely. This study was conducted to identify the viral coinfection prevalence among infected people with SARS-CoV-2.

## 2. Methods

We performed this study according to PRISMA guidelines [12]. Our study design was registered in the International Prospective Register of Systematic Reviews with CRD42021240030 registration number. We identified all of the studies which had our desired keywords including Coronavirus, COVID-19, SARS-CoV-2, SARS-CoV-2 infection, Polymicrobial Infection, Viral AND co-infections, Viral AND Secondary Infections, and Viral AND Mixed Infections.

### 2.1. Literature Search Method

An in-depth analysis of search engines, including Scopus, WOS, Medline, and Embase, was conducted to find relevant research papers on the viral coinfections among COVID-19-infected persons from December 1, 2019, to December 30, 2020. Preprint servers including medRxiv were also screened and data were retrieved. The authors selected keywords based on MeSH terms. Inclusion and exclusion criteria were defined using the PICO algorithms. We also designed our search strategy based on PICO algorithms. We considered both text words and MeSH terms to define our search keywords and their combinations were used to find the relevant articles.

A virologist identified the relevant articles. We send all of the related articles to Endnote X6. Afterward, we removed the duplicate articles. The remaining articles were reviewed in three steps. In the first step, we reviewed the title of the article and then the abstract, and finally, the article full texts were evaluated. Two authors reviewed the articles independently using these steps. The opinion of the third author was used to address the issues of “RR,” “SS,” and inter-rater discrepancies. We used both blinding and task separation procedures during the selection of the studies. The inter-rater agreement was 88%.

### 2.2. Inclusion and Exclusion Criteria

All of the related cross-sectional, case series studies, and cohort, were reviewed. The viral coinfection prevalence among patients was unrestricted. We excluded case series and case reports with less than 5 sample sizes, editorial, commentaries, case-control, and randomized clinical trials.

### 2.3. Data Extraction

In addition to general information, such as the name of authors, country, year, area, design of the study, COVID-19 patients numbers or sample size or, gender and age, other information, such as viral co-infections numbers and types, were extracted from all studies. We also included the COVID-19 patients (cases with positive COVID-19), and in addition, we even considered one viral coinfection in our study.

### 2.4. Subgroup Definition

We classified the viruses based on their transmission and clinical features. We categorized the countries according to the latest WHO guideline which contains six regions: Africa (AFRO), Americas (AMRO), Eastern Mediterranean (EMRO), Europe (EURO), South-East Asia (SEARO), and Western Pacific (WPRO).

### 2.5. Quality Assessment

The Newcastle-Ottawa Scale was applied to evaluate the quality of selected studies [13]. Two authors reviewed the articles separately, and the total score of each of the articles was calculated. Then, all of the selected studies were categorized based on these levels: very good, good, satisfactory, and unsatisfactory [14].

### 2.6. Statistical Analysis

We performed the analysis using Stata software 14.0. COVID-19 cases, viral coinfection prevalence, and types of viruses were extracted [14–18]. Cochran's *Q* test was used to determine the heterogeneity. We also applied the *I*^2^ index to quantify the heterogeneity. We considered the *I*^2^ value above 0.7 as high heterogeneity based on Higgins classification. The “meta pop” command was applied to evaluate the pooled prevalence with a 95% confidence interval (CI), and we used a random effects plot to discover the pooled prevalence. We used the meta-regression approach to evaluate the age, WHO region, and sample size effect on the study heterogeneity. Publication bias was checked by the “metabias” command. We adjusted the prevalence rate with the “metal trim” command to avoid any publication bias. A statistical significance of 0.05 was considered during the analysis.

## 3. Result

**Figure 1:**
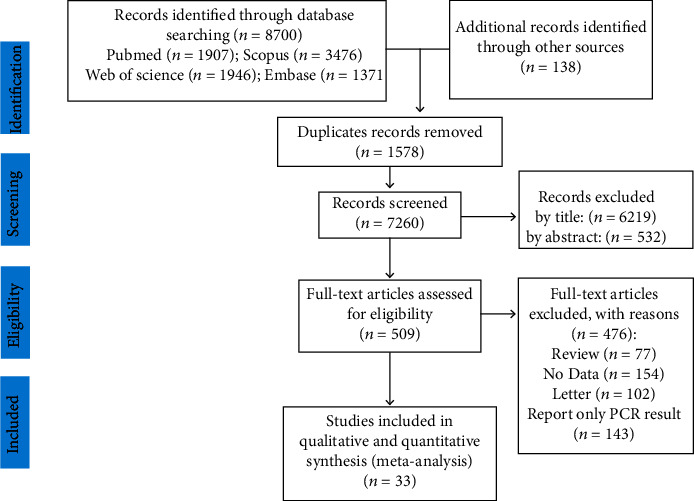
Study selection process PRISMA flow chart.

We found 8838 articles during the search process through scientific databases. Afterward, redundant papers were excluded and 7260 studies remained. We screened the articles in three independent steps. First, article titles were scanned: 6219 articles were excluded and 1041 studies remained. In the second step, the abstract was reviewed; we excluded 532 studies and in the final step; the full texts of the 509 remaining studies were scanned thoughtfully, and 476 studies were excluded. In the end, 33 studies [8, 19–49] with a total sample size of 10484 patients were analyzed.  Figure 1 exhibits this selection procedure, and the study characteristics are available in Table 1 and supplement 1. The maximum number of articles was from the Western Pacific Region (16 studies), and the Eastern Mediterranean area had the minimum number of studies (2 studies). The publication date of all the articles was 2020. The highest and lowest patient age was recorded by Hughes (mean age = 69.5) and Wu (mean age = 6), respectively. It should be mentioned that 25 (75.76%) of the articles were case series, 5 (15.15%) cohorts, and 3 (9.09%) cross-sectional.

### 3.1. Viral Coinfection Pooled Prevalence among COVID-19 Patients

**Figure 2:**
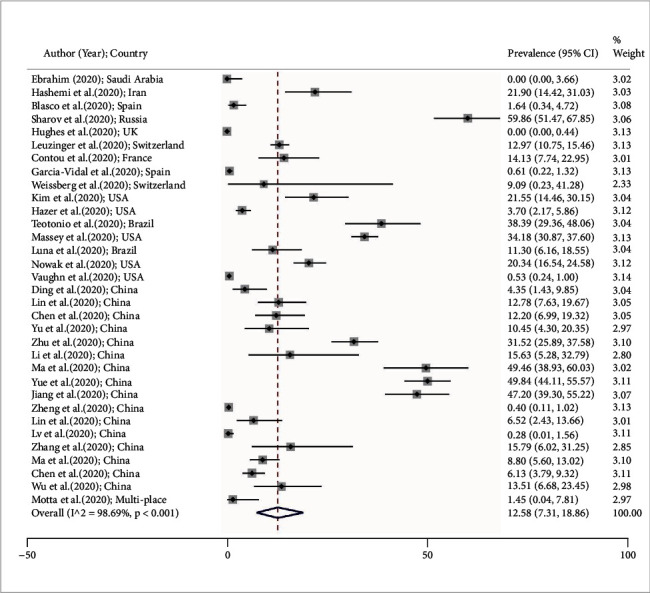
Forest plot shows prevalence of viral coinfections among COVID-19 patients according to the random effects approach. Every single article demonstrated by the first author (year) and country. Each line segment's midpoint exhibited the prevalence estimation, the line segment length presents 95% confidence interval (CI) in every study, and the diamond mark points out the pooled estimation.

We presented the viral coinfection prevalence in Table 1.  Figure 2 shows the viral coinfection prevalence forest plot. Ebrahim in Saudi Arabia reported the lowest viral coinfection prevalence (prevalence: 0%; 95% CI: 0 to 3.66) [25], and the highest levels of coinfection prevalence (prevalence: 59.86; 95% CI: 51.47 to 67.85) [39] were recorded by Sharov in Russia. By performing a random effects model, the pooled estimated prevalence of viral coinfections was calculated at 12.58% (95% CI: 7.31 to 18.96) (Figure 2). Our results indicated that from every 1000 COVID-19 patients, 73 to 190 individuals had viral coinfections.

### 3.2. Pooled Prevalence of Viral Coinfections Based on Different Subgroups

**Figure 3:**
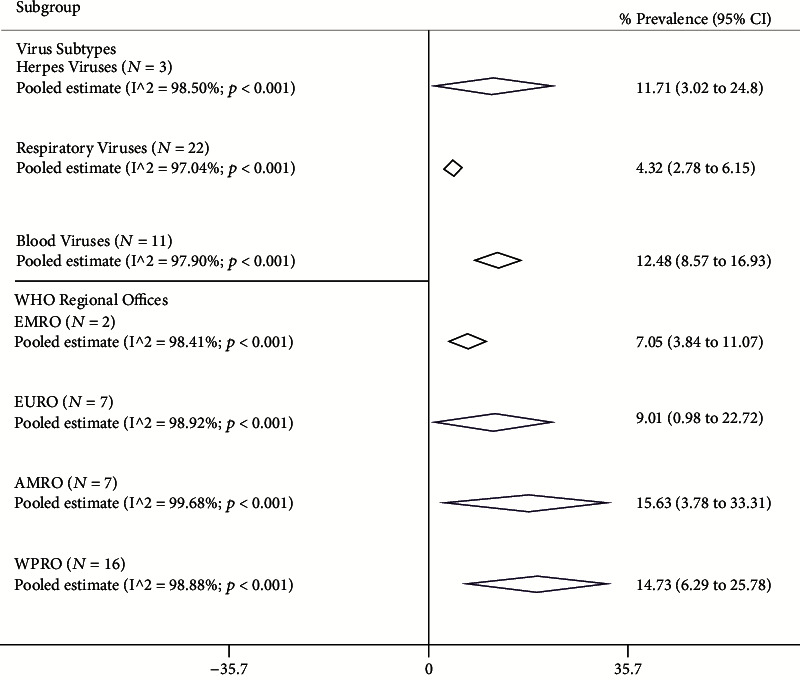
Pooled prevalence with 95% confidence interval (CI) and heterogeneity indexes of viral coinfections among the COVID-19 patient based on the virus type and different region. The diamond mark exhibits the pooled prevalence and the diamond length shows 95% CI.

The viral coinfection pool prevalence is available in Figure 3 according to virus subtypes and the area of the study. The most frequent subtype of viral coinfections was blood viruses (pooled prevalence: 12.48%; 95% CI: 8.57 to 16.93), and the less frequent virus subtype was respiratory viruses (pooled prevalence: 4.32%; 95% CI: 2.78 to 6.15). The herpesvirus pooled prevalence was 11.71% (95% CI: 3.02 to 24.80). Also, the most and least pooled prevalence of viral coinfections was estimated in AMRO and EMRO with 15.63% (95% CI: 3.78 to 33.31) and 7.05% (95% CI: 3.84 to 11.07), respectively. The EURO and AMRO pooled prevalence is shown in Figure 3.

### 3.3. Heterogeneity and Meta-Regression

**Figure 4:**
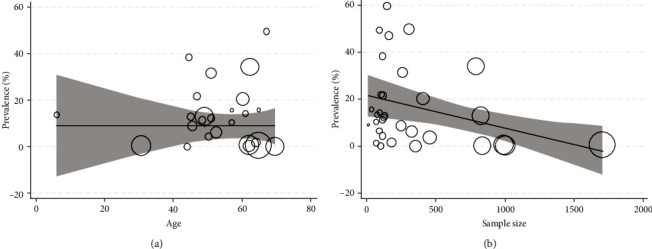
Association among (a) age prevalence and (b) sample size with viral coinfection prevalence by applying meta-regression. The circle size shows each study's precision. There is no considerable association with respect to the viral coinfection prevalence with age sample size.

Heterogeneity exported data are available in Table 2. Cochran's heterogeneity *Q* test exhibited that heterogeneity was significant in the articles (*P* < 0.001). The *I*^2^ index total viral coinfections and their different subtypes were up to 90%. Meta-regression results also identified that the age (coefficient: 2 × 10^−4^; *P* = 0.708), sample size (coefficient: −1 × 10^−4^; *P* = 0.152), and WHO region size (coefficient: 2.225; *P* = 0.605) were not effective on heterogeneity (Figures 4(a) and 4(b)).

### 3.4. Publication Bias

Egger's test showed that there wasn't considerable publication bias in our study.

## 4. Discussion

Our review revealed that most studies about viral coinfection among COVID-19 patients were conducted in the Western Pacific area. The Eastern Mediterranean region recorded the lowest number of studies. The lowest coinfection prevalence was in Saudi Arabia, and the highest prevalence has been reported in China. The highest rate of coinfection related to blood viruses was 12.48%, and the lowest rate of coinfection belonged to respiratory viruses. The highest coinfection prevalence and the lowest coinfection prevalence were in WPRO and EMRO, respectively. Respiratory syncytial virus (RSV) is the main diagnosed respiratory virus among COVID-19-infected persons. A systematic review showed that RSV is a commonly isolated respiratory virus from different age groups and is the lower respiratory tract infection (LRTI) main causative agent in young children. Respiratory viruses are cross-species transmittable and exhibit close clinical symptoms to COVID-19, which turns them into a potential threat to people infected with COVID-19 [50, 51].

Respiratory viruses may remain infectious and continue to circulate and cause coinfection during new respiratory outbreaks; however, their coinfection with COVID-19 is estimated to be 0–3%. However, recent studies suggested a higher coinfection incidence with other respiratory viruses [51, 52].

It is difficult to accurately estimate the prevalence of SARS-CoV-2 coinfection with other types of respiratory viruses. Their similar nature and common clinical feature, as well as the lack of enough diagnostic equipment, are the major factors that limit us to distinguish them from each other. Therefore, it can be expected that the prevalence of these viruses among the patients is higher than those of the previous reports [53].

Another study showed that 11.6% of COVID-19 patients had coinfection. This study concluded that coinfection with respiratory viruses is common among COVID-19 patients [52].

Accurate estimation of the respiratory virus's coinfection prevalence rate among infected people with SARS-CoV-2 will lead to a better understanding of their role in the disease and also improve the diagnosis and course of treatment [54]. Our results indicated that the highest prevalence of coinfection belonged to blood-borne viruses (BBVs). Our results are in contrast with other studies which reported RSV as the most common virus among COVID-19 patients [35, 52].

There are only a limited number of studies about SARS-CoV-2 and HIV coinfection [55]; some people living with HIV (PLHIV) and especially males affected by ARV-related complications could be more prone to severe COVID-19 disease [56]. The provided data about PLHIV showed that older patients may have poor morbidity and mortality condition with SARS-CoV2-HIV coinfection. A systematic review found that COVID-19-HIV patients had comorbidities with hypertension with 39.3% and 19.3% for obesity or hyperlipidemia, 18.0% had chronic obstructive pulmonary disease, and 17.2% of them had diabetes, and the majority of these patients were males over 50 years old [57, 58].

Coinfection between hepatitis viruses and SARS-CoV-2 is also quite controversial. SARS-CoV-2-HBV cases showed 4.7% and 15% mortality rates in cross-sectional and case report studies, respectively. SARS-CoV-2-HCV cases had an 8.3% mortality rate [59]. Regardless of infection with these two viruses, these patients had at least one comorbidity factor, including type 2 diabetes and hypertension. Liver enzyme abnormalities and acute hepatic injuries were observed in HBV and HCV patients, although it is yet unknown that the liver damage in these patients is related to COVID-19 or the hepatitis viruses and the interaction between these viruses and SARS-CoV-2 [60, 61]. Another study found that the liver damage prevalence among COVID-19 patients was 4% and caused by HBV. However, the study stated that there wasn't any relationship between COVID-19 coinfection in patients with chronic hepatitis and increased mortality rate.

In contrast to that, another research concluded that there are a considerable risk of mortality and morbidity between infected people with SARS-CoV-2 and HBV and HCV [59, 62]. Our result indicated that the highest rate of coinfection with COVID-19 belongs to blood-borne viruses. This result may happen because of a lack of enough research on respiratory coinfection with COVID-19 or low sample size in the studies on the subject. Another possible reason for this result could be the concurrent infection with the blood virus.

Immunocompromised patients are susceptible to viruses, especially herpes viruses, such as human HCMV and EBV. This virus reactivation in intensive care unit (ICU) patients is associated with their morbidity and mortality [63]. EBV DNA was observed in 95.2% of the ICU patients and 83.6% of the SICU patients infected with COVID-19 [64]. EBV reactivation is notably related to more prolonged ICU length of stay in COVID-19 patients [65]. COVID-19 patients show a reduction in NK and CD8+ T cells and the presence of EBV DNA. Also, a higher count of B cells in people with severe COVID-19 infection compared to patients with a mild form of the disease was observed [64]. The impact of an increase in EBV DNA on B-cell function is not pretty clear, but it can be postulated that there is a potential impact of the virus on COVID-19 severity. EBV has a distinguished role in COVID-19-infected cases compared to HCMV and HHV-6; among herpesviruses, only EBV reactivated in the patients. There wasn't a significant relationship between HHV-6 and CMV reactivation and clinical outcomes in COVID-19 patients.

Reactivation of EBV occurs in the early phase after patients being admitted to the ICU. It is evident that critically ill patients are vulnerable to latent viruses, especially EBV reactivation, and due to their relevance with morbidity and mortality in immunocompromised individuals such as ICU and transplanted patients, more research is needed to investigate their association with COVID-19 and their impact on the different outcome of the COVID-19 infection [64, 65].

Infections with rhinovirus were also investigated during COVID-19 pandemics. Kim et al. [8] showed that rhinovirus infection prevalence was 6.9%. The prevalence of nonpolio enterovirus activity decreased due to the COVID-19 pandemic and nonpharmaceutical interventions. For example, a lower incidence of NPEV was observed during the 2019–2020 season in Taiwan [8, 66]. The prevalence of viral coinfection with COVID-19 may be affected by other viral outbreaks, such as influenza virus outbreaks and different anti-COVID measurements like nonpharmaceutical measurement and its effect on enterovirus activity in Taiwan.

There was high heterogeneity between the coinfection prevalence in different countries (*I*^2^ = 98%), which indicates that a large part of the calculated total variance in our study was because of the differences between these studies. Even combining the results of these researches with the random effects method was not helpful, and the metaregression method could not find the heterogeneity causative agents. Therefore, the mean age, sample size, and study location had no effect on heterogeneity among the studies. Also, there wasn't a similar meta-analysis study that evaluated different variable's effects on heterogeneity. We found only a few preliminary studies that evaluated the effects of different variables such as gender, length of stay, and hospitalization in the ICU [49, 55, 56, 58, 60, 67]. Due to the low number of studies and their similar results, using metaregression methods was not applicable to investigate the variable impact.

There was also some limitation in our study: first, because there wasn't enough data, we could not perform the gender-specific estimation. This means that the number of studies reporting the gender prevalence was limited and we could not calculate robust gender pooled prevalence.

Second, we tend to calculate the pooled prevalence based on WHO territorial office data and perform spatial analysis in several geographical districts to distinguish the high-risk area for viral coinfection [68–76], but this estimation may be unreliable because of the small number of studies.

The current study's strengths were conducting a comprehensive search using various strategies such as searching online databases and preprint servers, manual searching to detect related articles, and estimating the pooled prevalence based on different subtypes of viruses and WHO regions.

## 5. Conclusion

Our study demonstrated that COVID-19 coinfections are prevalent among infected patients with blood-borne viruses (BBVs) such as HIV or HCV and surprisingly, the lowest rate of coinfection belonged to respiratory viruses. Due to the prevalence of influenza virus or RSV among societies, more studies need to be done to clarify the incidence rate of viral coinfection in COVID-19 patients. Another important aspect that should be investigated is their relationship with the morbidity and mortality of the patient. In a nutshell, further investigation about viral coinfection with SARS-CoV-2 is an urgent need.

## Figures and Tables

**Table 1:** Evaluated articles featured in the present meta-analysis.

Author	Country	Design	Publication year	Mean age	Sample size	Viral coinfection prevalence (95% CI)
Zhu et al. [48]	China	Retrospective case series	2020	51	257	31.52 (25.89 to 37.58)
Zheng et al. [47]	China	Retrospective case series	2020	30.6	1001	0.40 (0.11 to 1.02)
Blasco et al. [19]	Spain	Retrospective case series	2020	64	183	1.64 (0.34 to 4.72)
Contou et al. [22]	France	Retrospective case series	2020	61	92	14.13 (7.74 to 22.95)
Chen et al. [20]	China	Retrospective case series	2020	52.5	326	6.13 (3.79 to 9.32)
Chen et al. [21]	China	Retrospective case series	2020	51	123	12.20 (6.99 to 19.32)
Luna et al. [23]	Brazil	Case series	2020	48.49	115	11.30 (6.16 to 18.55)
Ding et al. [24]	China	Case series	2020	50.2	115	4.35 (1.43 to 9.85)
Ebrahim [25]	Saudi Arabia	Case series	2020	44	99	0.00 (0.00 to 3.66)
Garcia-Vidal et al. [26]	Spain	Retrospective case series	2020	62	989	0.61 (0.22 to 1.32)
Hashemi et al. [27]	Iran	Case series	2020	—	105	21.9 (14.42 to 31.03)
Hazra et al. [28]	Chicago	Cross-sectional	2020	—	459	3.70 (2.17 to 5.86)
Hughes et al. [29]	UK	Retrospective case series	2020	69.5	836	0.00 (0.00 to 0.44)
Jiang et al. [30]	China	Case series	2020	≤14	161	0.40 (0.11 to 1.02)
Kim et al. [8]	California	Cross-sectional	2020	46.9	116	21.55 (14.46 to 30.15)
Leuzinger et al. [31]	Switzerland	Prospective case series	2020	49	825	12.97 (10.75 to 15.46)
Li et al. [49]	China	Case series	2020	57	32	15.63 (5.28 to 32.79)
Lin et al. [32]	China	Retrospective case series	2020	18-65	92	6.52 (2.43 to 13.66)
Lin et al. [33]	China	Retrospective case series	2020	45	133	12.78 (7.63 to 19.67)
Lv et al. [34]	China	Retrospective cohort	2020	62	354	0.28 (0.01 to 1.56)
Ma et al. [35]	China	Case series	2020	45.5	250	8.80 (5.60 to 13.02)
Ma et al. [36]	China	Cross-sectional	2020	67	93	49.46 (38.93 to 60.03)
Massey et al. [67]	USA	Retrospective case series	2020	62.3	790	34.18 (30.87 to 37.60)
Motta et al. [37]	Multiplace^∗^	Cohort	2020	—	69	1.45 (0.04 to 7.81)
Nowak et al. [38]	New York	Retrospective case series	2020	60.2	408	20.34 (16.54 to 24.58)
Sharov et al. [39]	Russia	Retrospective case series	2020	—	147	59.86 (51.47 to 67.85)
Teotonio et al. [40]	Brazil	Retrospective case series	2020	44.55	112	38.39 (29.36 to 48.06)
Vaughn et al. [41]	Michigan	Cohort	2020	64.7	1705	0.53 (0.24 to 1.00)
Weissberg et al. [42]	Switzerland	Retrospective cohort	2020	49	11	9.09 (0.23 to 41.28)
Wu et al. [43]	China	Retrospective case series	2020	6	74	13.51 (6.68 to 23.45)
Yu et al. [44]	China	Prospective cohort	2020	57	67	10.45 (4.30 to 20.35)
Yue et al. [45]	China	Retrospective case series	2020	—	307	49.84 (44.11 to 55.57)
Zhang et al. [46]	China	Retrospective case series	2020	64.76	38	15.79 (6.02 to 31.25)

CI: confidence interval; ^∗^Belgium, Brazil, France, Italy, Russia, Singapore, Spain, and Switzerland.

**Table 2:** The univariate meta-regression analysis on the determinant heterogeneity in viral coinfections among COVID-19 patient studies.

Variables	Coefficient	95% CI	*P* value
Age (year)	2 × 10^−4^	−4 × 10^−3^ to 5 × 10^−3^	0.897
WHO region (score)	2.225	-6.317 to 11.404	0.598
Sample size (number)	−1 × 10^−4^	−27 × 10^−5^ to 2 × 10^−5^	0.090

CI: confidence interval; coding of WHO region: 1: EMRO; 2: EURO; 3: AMRO; 4: WPRO.

## Data Availability

All data can be obtained from corresponding authors Abbas Farahani and Saber Soltani or the co-first authors Pooneh Malekifar and Reza Pakzad.

## Supplementary Materials

Supplementary Materials Supplement 1: type of virus coinfection in included studies.
