# Supplementary material for: Viral Coinfection among COVID-19 Patient Groups: An Update Systematic Review and Meta-Analysis
Source: Biomed Res Int. 2021 Sep 3;2021:5313832. doi: 10.1155/2021/5313832 (PMC8416381; doi:10.1155/2021/5313832)
Supplement: Supplementary Materials — Supplement 1: type of virus coinfection in included studies. [file 5313832.f1.docx]

**Supplement 1:** type of virus coinfection in included studies

| I**D** | **Author** | **Respiratory Viruses** | **Blood viruses** | **Herpes viruses** |
| --- | --- | --- | --- | --- |
| 1 | Zhu et al.(48) | Human rhinovirus: (4.7%); Influenza virus B: (1.9%); Influenza virus A: (0.8%); human boca virus adenovirus: (0.4%); human metapneumo virus: (0.4%); human adenovirus: (3.9%) | --- | Epstein–Barr virus: (20.2%); herpes simplex virus: (3.1%);  Cytomegalovirus: (1.2%) |
| 4 | Zheng et al.(47) | Influenza virus: (0.4%) |  |  |
| 29 | Blasco et al.(19) | Adenovirus: (0%); Human bocavirus: (0%); Human metapneumovirus: (0%); Influenza virus A: (0%); Parainfluenza virus 3: (0%); Respiratory syncytial virus (A/B): (0%); Rhinovirus/Enterovirus: (0%) | --- | --- |
| 37 | Contou et al.(22) | Influenza virus A and B: (14%) |  | --- |
| 45 | Chen et al.(20) |  | Hepatitis-B: (6.1%); Hepatitis-B: (6.1%) | --- |
| 48 | Chen et al.(21) |  | Hepatitis-B infection: (12%) | --- |
| 67 | Luna et al.(23) | Influenza viruses: (3%); respiratory syncytial virus: (3%); IV-A and human metapneumo virus: (0%) | --- | --- |
| 71 | Ding et al.(24) | Influenza virus: (4%) | --- | --- |
| 92 | Garcia-Vidal et al.(26) | Influenza virus A: (0.4%); Influenza virus B: (0.2%) | --- | --- |
| 103 | Hashemi et al.(27) | Influenza virus A (H1N1): (22.3%); metapenovirus: (2.9%); bocaviruse: (9.7%); Adenovirus: (1.9%); para influenza virus: (3.9%); respiratory syncytial virus: (9.7%); influenza virus: (22.3%) | --- | --- |
| 105 | Hazra et al.(28) | Rhinovirus– influenza virus A: (2.1%); coronavirus NL63: (2.1%); human metapneumovirus: (2%); Adenovirus 2: (0.4%); Coronavirus HKU1: (0%); Coronavirus NL63: (0.2%);  Coronavirus 229E: (0%); Coronavirus OC4: (30%); Human metapneumovirus: (0.4%); Influenza virus A: (0.7%); Influenza virus B: (0%); Influenza virus 1: (0%); Parainfluenza virus2: (0.2%); Parainfluenza virus 3: (0%); Parainfluenza virus 4: (0%); Rhinovirus/Enterovirus: (1.7%) | --- | --- |
| 115 | Hughes et al.(29) | Influenza virus: (0%) | --- | --- |
| 119 | Jiang et al.(30) | Human respiratory syncytial virus: (31.8%); Influenza virus A virus: (30.13%) | --- | --- |
| 133 | Kim et al.(8) | Rhinovirus/Enterovirus: (6.9%); respiratory syncytial virus: (5.2%); Influenza virus A: (0.9%); Influenza virus B : (0%); respiratory syncytial virus: (5.2%); Parainfluenza virus 1: (0.9%); Parainfluenza virus 2: (0%); Parainfluenza virus3: (0.9%); Parainfluenza virus 4 : (0.9%); Metapneumo virus: (1.7%);  Adenovirus: (0%); Other Coronaviridae: (4.3%) | --- | --- |
| 147 | Leuzinger et al.(31) | Rhinovirus/Influenza virus: (12%); coronavirus: (9%); respiratory syncytial virus: (6%); metapneumovirus: (6%) | --- | --- |
| 150 | Li et al.(49) | Respiratory syncytial virus: (3%); human parainfluenza virus: (3%); human metapneumovirus: (3%); Rhinovirus: (3%) | --- | --- |
| 154 | Lin et al.(33) |  | Hepatitis-B: (17%) | --- |
| 164 | Ma et al.(35) | Adenovirus: (2.8%); Influenza Virus A: (0.8%); Influenza Virus B: (0.4%); Respiratory Syncytial Virus: (4.8%) | --- | --- |
| 165 | Ma et al. (36) | Influenza virus: (49.6%) | --- | --- |
| 178 | Massey et al.(50) |  | --- | Epstein–Barr virus: (34.1%); HHV6: (34%) |
| 194 | Motta et al.(37) |  | HIV and Hepatitis-B / Hepatitis-D: (1%);  HIV and Hepatitis-B / Hepatitis-D: (1%);  HIV and Hepatitis-B / Hepatitis-D: (1%) | --- |
| 209 | Nowak et al.(38) | Influenza virus: (0.08%); Influenza virus A: (0.08%); Influenza virus B: (0%); Respiratory syncytial virus: (0.31%); Other Coronaviridae: (1.54%); Coronavirus NL63: (0.63%); Coronavirus HKU1: (0.45%); Coronavirus 229E: (0.36%); Coronavirus OC43: (0.09%); Rhinovirus/Enterovirus: (0.73%);  Human metapneumovirus: (0.36%); Adenovirus: (0.18%); Parainfluenza virus: (0%); Parainfluenza virus 1: (0%); Parainfluenza virus 2: (0%); Parainfluenza virus 3: (0%); Parainfluenza virus 4: (0%); Influenza virus/respiratory syncytial virus: (2.99%); | --- | --- |
| 271 | Sharov et al.(39) | Haemophilus influenza virus: (11%); Influenza virus: (59.55%);  Influenza virus B: (23.63%); respiratory syncytial virus: (21.26%); metapneumoviruses: (42.42%) | --- | --- |
| 308 | Vaughn et al.(41) | Influenza virus A or B: (0.1%) | --- |  |
| 322 | Weissberg et al.(42) | Adenovirus serotype 4: (1%) | --- |  |
| 331 | Wu et al.(43) | Respiratory syncytial virus: (15.8%); influenza virus A&B: (5.3%) | --- | Epstein–Barr virus: (15.8%); cytomegalovirus: (15.8%) |
| 343 | Yu et al. (44) |  | Hepatitis-B: (7%) | --- |
| 344 | Yue et al.(45) | Influenza virus A: (49.8%); influenza virus B: (7.5%) | --- | --- |
